# Association between urinary sodium excretion and hard outcomes in non-dialysis chronic kidney disease patients

**DOI:** 10.1186/s12882-022-02911-7

**Published:** 2022-08-18

**Authors:** Cecília Malheiro Cury, Vanessa Burgugi Banin, Pamela Falbo dos Reis, Jacqueline Costa Teixeira Caramori, Pasqual Barretti, Luís Gustavo Modelli de Andrade, Luis Cuadrado Martin

**Affiliations:** Division of Nephrology, Department of Medicine, Botucatu Medical School, São Paulo University, Botucatu, Sao Paulo Brazil

**Keywords:** Chronic kidney disease, Sodium intake, Urinary sodium excretion, Renal failure

## Abstract

**Background:**

Restriction of sodium intake is routinely recommended for patients with chronic kidney disease (CKD). Whether or not sodium intake is associated with the progression of CKD and mortality remains uncertain. We evaluated the association between urinary sodium excretion (as a surrogate for sodium intake) with the occurrence of renal failure and mortality in patients with non-dialytic CKD.

**Methods:**

We conducted a retrospective study of patients followed at a CKD clinic care hospital from October 2006 to March 2017. Adult patients with non-dialytic CKD were included. Using a time-to-event analysis, we examined the association of urinary sodium excretion as a categorical variable (categorized as quintiles: 1st quintile: 0.54–2.51 g; 2nd quintile: 2.52–3.11 g, 3rd quintile: 3.12–3.97 g, 4th quintile: 3.98–5.24 g and 5th quintile: 5.26–13.80 g) and the outcomes of interest. The primary outcome was defined as progression to end-stage renal disease requiring any type of renal replacement therapy. The secondary outcome was mortality.

**Results:**

Two hundred five patients were included in the study (mean follow up of 2.6 years) with a mean eGFR of 26 (19–41) ml/min/1.73m2. 37 patients (18%) required renal replacement therapy and 52 (25,3%) died. There was association between urinary sodium excretion and need for renal replacement therapy (adjusted HR 0.245; 95%CI 0.660–0.912). There was no association between urinary sodium excretion and mortality in adjusted models.

**Conclusion:**

Moderate sodium intake was associated with a lower risk of renal failure.

## Background

Chronic kidney disease (CKD) affects about 10% of the adult population [1], which is associated with an increased risk of renal replacement therapy, as well as cardiovascular morbimortality [2–5]. The CKD progression is understood as the loss of renal function, regardless the underlying disease and its activity [6].

The treatment aims is attenuate the progression of CKD interventions on modifiable factors: to increase survival, preparing the patient for a possible renal replacement therapy, and improving the quality of life [2]. Clinical strategies were directed at controlling blood glucose, arterial hypertension [7], dyslipidemia [8], obesity [9], smoking [10] and lifestyle adjustment. Dietary interventions play an important role and nutritional support is necessary in all stages of CKD [11], with special attention to the proteins [12], potassium [13], phosphorus [14] and sodium intake [14].

Current guidelines recommend a sodium consumption of 2.4 g/day for the adult population [15–17]. The emphasis in this recommendation is due the direct relationship with the increase in blood pressure [18–23]. In CKD patients, an excessive sodium intake is also associated with proteinuria [23, 24], besides attenuating the antihypertensive and anti-proteinuric effects of drugs acting on the renin-angiotensin system [25].

These mechanisms suggest the relationship between sodium intake with renal outcomes and mortality should be linear. Although several studies have shown association between increased sodium intake with CKD progression [13, 24, 26] and mortality [13, 26, 27], an inverse relationship has been reported by others [28].

In view of the heterogeneity of the published results, the aim of our study was determine if urinary sodium excretion is associated with mortality and need for renal replacement therapy in patients with non-dialytic CKD.

## Methods

### Patient population and measurements

This is a retrospective cohort study using prospectively collected data on adult patients (> 18 years) followed in the progressive renal insufficiency clinic at Botucatu Medical School, located in Botucatu, Brazil. The progressive renal insufficiency clinic is a specialty multi-disciplinary care clinic in the CKD program for patients approaching ESRD. Patients with progressive kidney disease are referred to this clinic at the discretion of primary nephrologist in anticipation of needing renal replacement therapy. Causes of CKD were: hypertension (40%), Diabetes (17,5%), Glomerular (5,9%) and others (pyelonephritis, ischemic nephropathy, nephrectomy, nephrocalcinosis, renal lithiasis, disease polycystic, undetermined cause). Attempts were made to collect 24-h urine for sodium excretion at least twice per year. Standardized instructions are given to each patient for collection of 24-h urine. The study included patients with non-dialytic CKD, at least two visits to the clinic and at least one 24-h urine sodium excretion (USE) measurement between October 2006 and November 2010. Patients who required any kind of renal replacement therapy prior to the laboratory test, in malignant phase of hypertension, hepatic insufficiency, alcoholics and with malignant neoplasm were excluded.

### Data collection

Data on all patients were obtained through the clinic’s comprehensive database since its inception in October 1st, 2006. The accuracy of the data in the database is verified every six months by auditing 5% of entries and accuracy has been > 95%. Data collected included age, gender, blood pressure (BP), body-mass index (BMI), eGFR, urine excretion of sodium, protein and creatinine, cause of CKD, comorbidities, medications, date of death and date of initial dialysis or renal transplantation.

Blood pressure was measured in sitting position after at least five minutes of rest. Blood pressure, eGFR and proteinuria were measured at each clinic visit. 24-h USE was performed periodically to monitor compliance and assist in counseling patients regarding sodium restriction. Hypertension was defined as an average BP ≥ 140/90 mmHg or self-reported use of antihypertensive drugs. Diabetes mellitus was defined according to the American Diabetes Associations criteria [29]. The estimated glomerular filtration rate (eGFR) was calculated according to the CKD-EPI Eq. (12).

Sodium intake for each patient was estimated from the average 24-h USE measured during the follow-up period. A priori, we categorized the patients according to their mean 24-h USE into five groups (*n* = 41):1st quintile: 0.54–2.51 g; 2nd quintile: 2.52–3.11 g, 3rd quintile: 3.12–3.97 g, 4th quintile: 3.98–5.24 g and 5th quintile: 5.26–13.80 g. The 1st quintile: (0.54–2.51 g) was selected as reference. We choose this point of sodium intake to be the recommended for patients CKD [15–17].

### Outcomes

The primary outcome was defined as progression to ESRD requiring any type of renal replacement therapy (dialysis or renal transplant). The secondary outcome was mortality from any cause. Surviving or of dialysis-free patients at the end of the follow, as well as those transferred from the service or having lost the segment were censored.

### Statistical analyses

Continuous variables are reported as mean (± SD) and categorical variables are reported as median interquartile range and percentages. Continuous variables were obtained by calculating the average between the results of the first and second exams. Chi squared test was used to compare categorical variables. Student’s “t” test and Mann Whitney test for non-parametric data. Time-to-event analyze was conducted using the Cox proportional hazards. Sodium intake was insert time-dependent Cox models using the quintiles 24-h USE. Those variables associated with outcomes in the preliminary analysis (level of 0.05) were used model Cox. Potential confounders included in the Cox models were age, smokers, eGFR, urinary protein/creatinine excretion. The variable PTH, serum phosphor, serum creatinine weren´t used in the Cox model because of collinearity with eGFR. Outcome mortality: age, eGFR, DBP, Diabetes e CVD were included in model Cox. For all tests, statistical significance was defined as *p* < 0.05.

## Results

Two Hundred Ninety-two patients were screened and 88 were excluded by lack or incomplete 24-h urine samples. The mean follow-up was 2.6 years. The baseline characteristics were presented in Tables 1 and 2. Two hundred five patients were included with a mean age of 64.8 years. Most patients were male (53,1%) and had a high proportion of whites (94,7%). At baseline, urinary protein excretion was 0,3 g/24 h and the mean eGFR was 26 ml/min/1.73 m2. The most common cause of CKD was hypertension. The majority of patients was hypertensive (97,4%) and 81,5% were in use of RAS blockade.

**Table 1:**
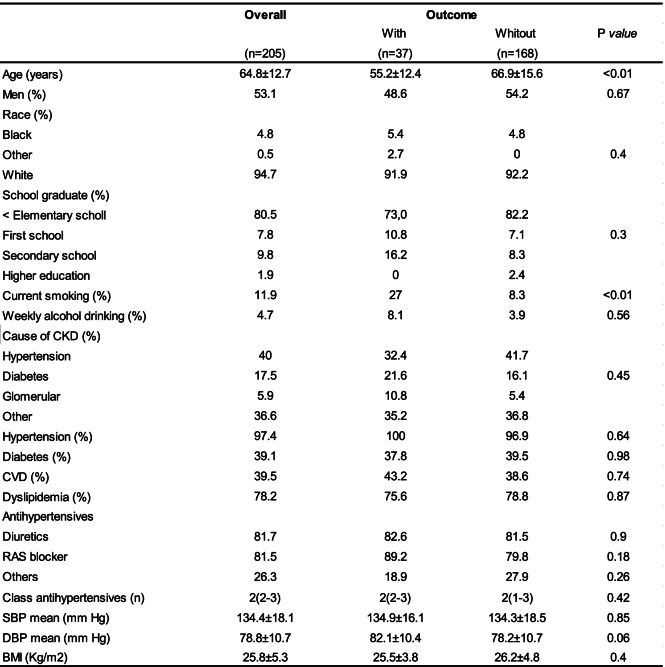
Demographic and clinical data of patients according to the outcomes renal

*CKD* Chronic kidney disease, *CVD* Cardiovascular disease, RAS blocker Blockers system renin-angiotensin, *SBP* Systolic blood pressure, *DBP* Diastolic blood pressure, *BMI* Body mass index

**Table 2:**
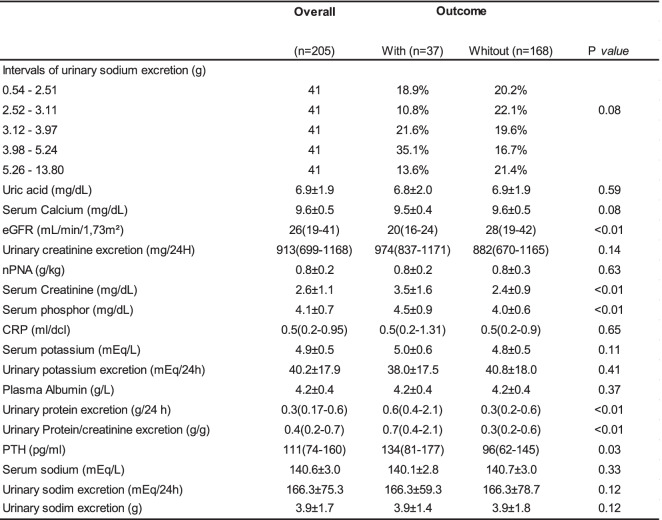
Laboratory data of patients according to the outcomes renal

*eGFR* estimated glomerular filtration rate, *npna*: protein nitrogen, *CRP* C-reative protein, *PTH* Parathyroid hormone

Based on the mean USE during the observational period, 205 patients were categorized into quintiles (*n* = 41) with the respective intervals: 1st quintile: 0.54–2.51 g; 2nd quintile: 2.52–3.11 g, 3rd quintile: 3.12–3.97 g, 4th quintile: 3.98–5.24 g and 5th quintile: 5.26–13.80 g. The average sodium daily intake of the participants was 3.9 g, which represents about 9.75 g of sodium chloride.

Thirty Seven patients (18%) reached the primary outcome.These patients had higher values of: serum creatinine, proteinuria, serum phosphor, urinary protein/creatinine excretion, parathyroid hormone and smokers. Age and eGFR were lower in patients who reached the primary outcome (Tables 1 and 2). The quintiles of USE showed marginal statistical differences in relation to the renal outcome (Tables 1 and 2).

**Fig. 1:**
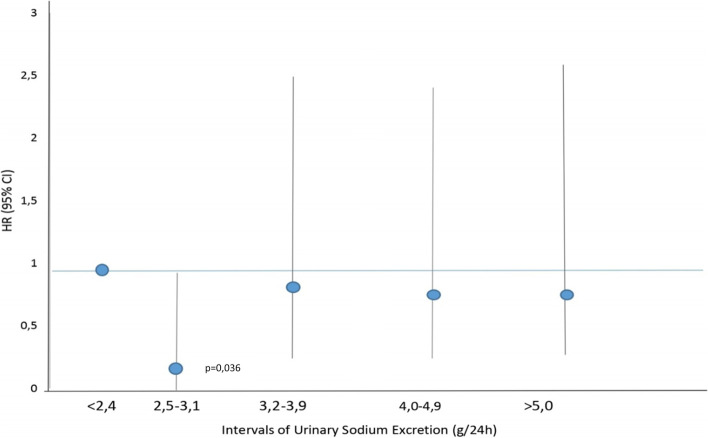
Association between urinary sodium excretion and risk renal failure (Cox Analyze). *Adjusted for age, eGFR smoker and urinary protein/creatinine excretion

In the adjusted Cox analysis that modeled USE as quintiles, 2nd quintile (2.52–3.11 g) was significantly associated with lower risk of progression to ESRD (HR 0.246; 95% CI 0.066–0.912); (Table 3). There were not associations between the others quintiles USE and primary outcome. Age, smokers, eGFR, urinary protein/creatinine excretion were included in the model Cox (Table 3). Variables that show collinearity with eGFR (PTH, serum phosphor, serum creatinine) were excluded from the model. This result lightly demonstrated a “U” shaped curve (Fig. 1).

**Table 3:**
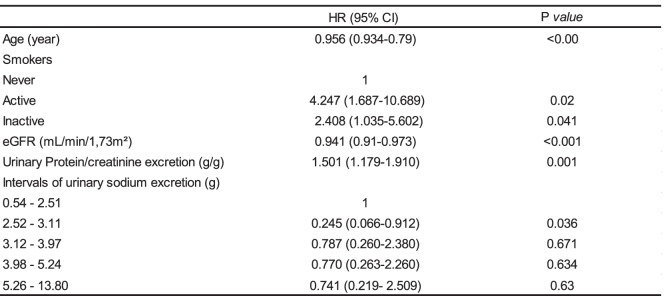
Time-dependent Cox model, according to the outcomes renal

*eGFR* estimated glomerular filtration rate

During the follow-up, 52 patients (25%) died. The causes of mortality were: 23 (44%) infectious disease, 18 (35%) CVD, five (10%) malignancies and six (12%) deaths to others causes. Variables that showed statistical differences in relation to the secondary outcome were: age, previous CVD, eGFR and urinary creatinine excretion (Tables 4 and 5).

**Table 4:**
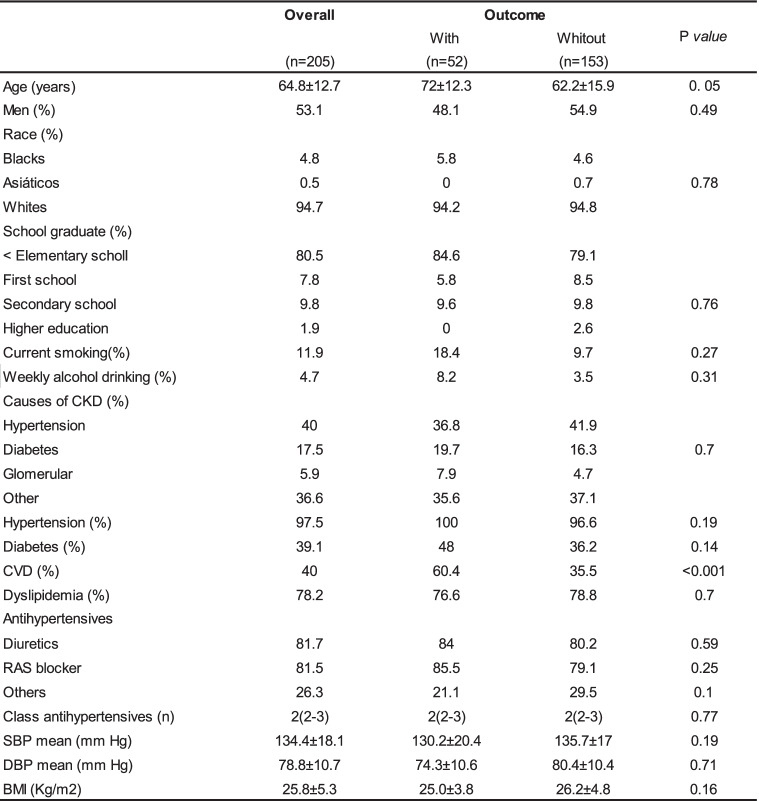
Demographic and clinical data of patients according to the outcomes death

*CKD* Chronic kidney disease, *CVD* Cardiovascular disease, *RAS blocker* Blockers system renin-angiotensin, *SBP* Systolic blood pressure, *DBP* Diastolic blood pressure, *BMI* Body mass index

**Table 5:**
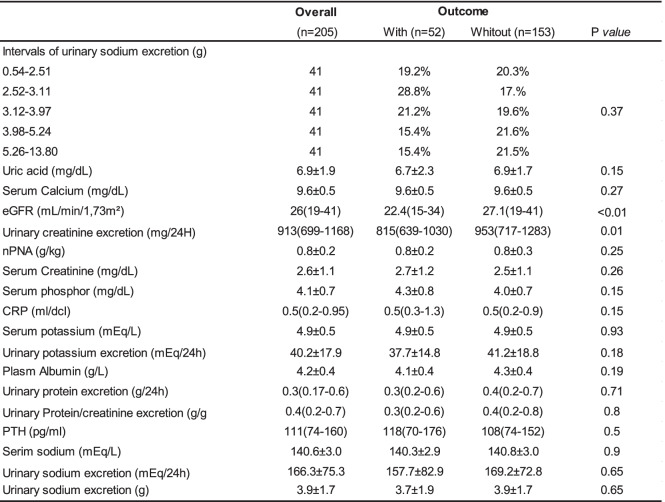
Laboratory data of patients according to the outcomes death

*eGFR* estimated glomerular filtration rate, *npna* protein nitrogen, *CRP* C- reative protein, *PTH* Parathyroid hormone

In the model Cox adjusted for: quintiles USE, age, eGFR, DBP, diabetes and DCV was not significant association with mortality. The variables DBP and diabetes, even without statistical significance in the preliminary analyzes, were included in the model to be considered risk factors to the outcome. Finally, only age achieved a statistically significant (HR 1.048; 95% CI 1.015–1.081); (Table 6).

**Table 6:**
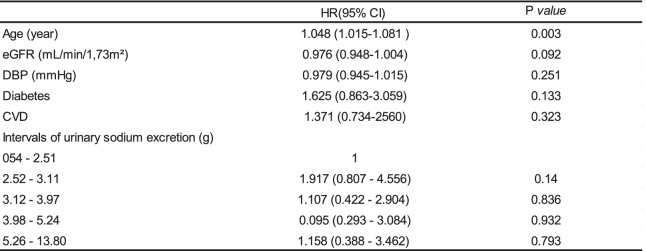
Time-dependent Cox model, according to the outcomes death

*eGFR* estimated glomerular filtration rate, *DBP* Diastolic blood pressure, *CVD* Cardiovascular disease

## Discussion

This study showed that urinary sodium excretion (as a surrogate for sodium intake) between 2.52–3.11 g/day was associated with a lower risk ESRD when compared with the reference 0.54 – 2.51 g/day. No other urinary sodium excretion quintile was associated with the primary outcome. Likewise, was not significant association with mortality.

Studies that evaluated the association between sodium intake and renal outcome obtained some different results [13, 24]. In the reanalysis of REIN-2 study, it was reported that sodium intake greater than 4.59 g/day was associated with increased risk of CKD progression [24]. This study suggests that sodium intake attenuates the antiproteinuric effect of angiotensin converting enzyme inhibitors therapy. However, when adding the proteinuria variable to the model, there was a loss of significance.

He et al. showed that urinary sodium excretion above 4.47 g was associated with 54% increase risk of CKD progression [13]. The cohort mentioned were composed by a high proportion of black patients, who classically have greater sodium sensitivity than whites. Our cohort was composed predominantly for whites.

In the studies cited above, higher sodium intakes were associated with CKD progression in models without the variable proteinuria. In the current study, the association between the second quintile of sodium and renal failure was obtained after adjustment for proteinuria. This association was independent of protein intake and BP.

In humans, a moderate restriction in sodium intake potentiates the antiproteinuric and antihypertensive effects of renin angiotensin system blockers [25]. However, others authors have proposed that daily sodium intakes lower than 2.0 g are associated with: vascular endothelial injury and inflammation [30]; rennin angiotensin system activation, increased levels of angiotensin II and NADPH oxidase and reactive oxygen species production [31], and sympathetic system activation [30]. Therefore, we could suggest that sodium moderate intake reduction attenuates the CKD progression, but an extreme restriction intake of this ion is not protective. Thus, an efficient strategy for practical recommendations is not prescribing extreme restrictions, which will compromise patients' quality of life.

An interesting finding of this study was that smokers evidenced greater risk to ESRD. This result corroborates to previous research [32, 33]. Experimental data showed that nicotine acts in the proximal tubule causing biosynthesis of pro-fibrotic and pro-inflammatory cytokines, which can accelerate the CKD progression [34], besides promotes deregulation of vasoconstrictor and vasodilator mediators [35] (39).

In relation to the mortality outcome, Mills et al. [26] reported that patients with established CVD and sodium intake above 4.54 g/day were associated with high risk for CVD [27]. In the present study, we did not obtain significant associations between the different quintiles of urinary sodium excretion and mortality (including cardiovascular causes). When comparing the two studies, Mills' study [26] also contained a higher proportion of: black, hypertensive, obese, smokers and diabetic patients. Factors risk known to cardiovascular mortality.

Our study should be interpreted taking into account its limitations. First, this is an observational study which does not allow definitive conclusions on the causality of the association between sodium intake and outcomes. Only two 24-h urine samples may not be enough to estimate the usual sodium intake with precision, but most studies have performed only a single dosage. As points positive: was added the proteinuria in the model Cox because is an important factor in CKD progression. In addition, we performed a measurement of sodium intake by the method of 24-h USE, which is considered the gold standard [36]. Finally, there were no previous Brazilian studies reporting an association between sodium intake with ESRD and mortality in non-dialysis CKD patients.

## Conclusion

In conclusion, in this longitudinal study, moderate sodium intake was associated with a lower risk of progression to renal failure in patients with established CKD. Further studies are needed confirm the association between sodium intake and outcomes in non-dialysis CKD patients.

## Data Availability

Due to ethical restrictions related to patient confidentiality, data are available after approval from the Committee of Botucat Medical School – Sao Paulo State University for researchers who meet criteria for access to confidential data. Questions regarding this process may be directed to Ms. Cury CM (cecilia.malheiro@unesp.br).

## Abbreviations

BMI: Body mass index
BP: Blood pressure
CKD: Chronic kidney disease
eGFR: Estimated glomerular filtration rate
g: Grams
ESRD: End-stage renal disease
RAS: Renin-angiotensin system
RRT: Renal replacement therapy
